# Detection of bacterial pathogens including potential new species in human head lice from Mali

**DOI:** 10.1371/journal.pone.0184621

**Published:** 2017-09-20

**Authors:** Nadia Amanzougaghene, Florence Fenollar, Abdoul Karim Sangaré, Mahamadou S. Sissoko, Ogobara K. Doumbo, Didier Raoult, Oleg Mediannikov

**Affiliations:** 1 Aix Marseille Univ, CNRS, IRD, INSERM, AP-HM, URMITE, IHU - Méditerranée Infection, Marseille, France; 2 University of Bamako, Epidemiology Department of Parasitic Diseases, Faculty of Medicine and Odonto-Stomatology, Faculty of Pharmacy (MRTC/DEAP/FMOS-FAPH), Bamako, Mali; 3 Campus International UCAD-IRD, Dakar, Senegal; Tianjin University, CHINA

## Abstract

In poor African countries, where no medical and biological facilities are available, the identification of potential emerging pathogens of concern at an early stage is challenging. Head lice, *Pediculus humanus capitis*, have a short life, feed only on human blood and do not transmit pathogens to their progeny. They are, therefore, a perfect tool for the xenodiagnosis of current or recent human infection. This study assessed the occurrence of bacterial pathogens from head lice collected in two rural villages from Mali, where a high frequency of head lice infestation had previously been reported, using molecular methods. Results show that all 600 head lice, collected from 117 individuals, belonged to clade E, specific to West Africa. *Bartonella quintana*, the causative agent of trench fever, was identified in three of the 600 (0.5%) head lice studied. Our study also shows, for the first time, the presence of the DNA of two pathogenic bacteria, namely *Coxiella burnetii* (5.1%) and *Rickettsia aeschlimannii* (0.6%), detected in human head lice, as well as the DNA of potential new species from the *Anaplasma* and *Ehrlichia* genera of unknown pathogenicity. The finding of several Malian head lice infected with *B*. *quintana*, *C*. *burnetii*, *R*. *aeschlimannii*, *Anaplasma* and *Ehrlichia* is alarming and highlights the need for active survey programs to define the public health consequences of the detection of these emerging bacterial pathogens in human head lice.

## Introduction

Humans are parasitized by three different types of sucking lice (*Anoplura*): the head louse, the body louse and the crab (pubic) louse, each of them colonizing a specific region of the body (head, body and pubic area, respectively) [1,2]. Two of these types are of great concern to public health and are now believed to be members of a single species, *Pediculus humanus*, which appears in two ecotypes *P*. *h*. *capitis* (known as the head louse) and *P*. *h*. *humanus* (also known as the body or clothing louse) [3,4]. Both ecotypes have the same life cycle and feed exclusively on human blood. They nevertheless occupy distinct ecological niches and have distinctly different feeding patterns [1,3,4]. Head lice live exclusively in the scalp region of humans, where the females lay eggs (nits) at the base of hair shafts [1,3]. They are prevalent worldwide, particularly in school-aged children, regardless of hygiene conditions and can cause very intense pruritus that may lead to high irritation and even wound infection [4–6]. In contrast, body lice feed on the body regions of humans and the females secure their eggs to clothing [1,3]. They were also very common in the past, but are more rarely encountered in modern times and tend to be restricted to precarious populations living in poor sanitary conditions, such as the homeless, war refugees and the prison population [4,5].

Head lice have been considered to be the ancestral lineage from which body lice have relatively recently emerged and probably on multiple occasions [3,5,7,8]. However, this claim is not supported by genomic analysis, as only one nuclear genetic marker has been identified based on variations in the PHUM540560 gene, which encodes a hypothetical 69-amino acids protein of unknown function that can unambiguously distinguish head from body lice once they are removed from their habitat. In body lice, this gene is present and expressed, whereas it is present but not expressed in head lice (deleted) [9]. In contrast, genetic studies based on mitochondrial DNA (mtDNA) appear to separate head lice into five divergent mitochondrial clades (A, B, C, D and E) and place body lice only in two clades, A and D [3,10,11], together with head lice. Clade A is the most common and spread worldwide, while clade D has to this point only been found in Africa [12]. Clade B is found in America, Europe, Australia, North and South Africa, and was most recently reported in Israel on head lice remains dating from approximately 2,000 years ago [11,13]. Clade C is found in Ethiopia, the Republic of Congo, the Asian continent and, recently, in France [3,12]. Lastly, the fifth clade, which has previously been described as sub-clade within clade C [12,14], and which has been only recently classified as a separate new clade is referred here as clade E [11]. This clade consists of head lice from West Africa (Senegal and Mali) [11,13]. All these data support the hypothesis that all current human lice travelled with archaic hominids (and slaves) from Africa.

Human body lice are the main vectors of three serious human pathogens: *Rickettsia prowazekii* (the causative agent of epidemic typhus), *Bartonella quintana* (trench fever) and *Borrelia recurrentis* (relapsing fever) [1,5]. There are natural and experimental observations that body lice can also transmit *Yersinia pestis*, the causative agent of plague, and that they may be the pandemic vectors of this agent [15–17]. Some other widespread pathogenic bacteria, such as *Serratia marcescens*, *Acinetobacter baumannii* and *A*. *lwoffii*, have been detected in human body lice with the assumption that lice can probably also transmit these agents to humans [4,18,19]. Early field observation in East Africa showed that human lice collected from a place where an epidemic of Q fever occurred three months previously, contained its agent, *C*. *burnetii*. Bacterial strains were recovered from these lice using guinea pigs [20]. Experimentally infected body lice are also capable of transmitting *R*. *typhi* (the causative agent of endemic or murine typhus), *R*. *rickettsii* (Rocky Mountain spotted fever) and *R*. *conorii* (Mediterranean spotted fever, Indian tick typhus) to rabbits [21,22].

Although body lice are much more potent vectors of pathogens than head lice, perhaps due to the size of the blood meal ingested (body lice ingest a larger blood meal) [5,23], and have played a principal role in all louse-borne outbreaks investigated through human history, this does not preclude head lice as additional vectors [24]. Moreover, in the last few decades, the status of head lice as a vector of pathogens has been raised, since body louse-borne pathogens have been increasingly detected in head lice collected worldwide, particularly in poor African countries, but also in the USA and France [4,10,12,14,25–27]. This is the case of *B*. *quintana* DNA found in head lice belonging to Clade A, E, C and D [6,10,14,25,28,29]. Other pathogens, such as *B*. *recurrentis*, *Y*. *pestis* and several *Acinetobacter* species have also been detected in human head lice [12,29–31]. Furthermore, experimental infections with *R*. *prowazekii* have shown that head lice can be readily infected and disseminate these pathogen in their feces, demonstrating that these lice have the potential to be a vector pathogen under optimal epidemiologic conditions [24].

Emerging infectious diseases represent a challenge for global economies and public health. In remote and underdeveloped regions of the African continent, often no attention is paid towards possible cases of infectious disease until a threshold of serious cases and deaths appears in a cluster and certain epidemic properties are reached. Lice infestations always occurs through blood meals, and the louse remains infected for its entire short life (one month), witnessing a recent human infection [1,32].The usefulness of PCR in detecting bacterial DNA in lice has been demonstrated by several investigations. Furthermore, several reports have demonstrated that the study of lice and associated pathogens can be used to detect infected patients (xenodiagnosis), estimate the risk for outbreaks, follow the progress of epidemics, and justify the implementation of control measures to prevent the spread of infection [32,33]. Our laboratory’s experience in Burundi is the best example of this. Thus, in 1995, D. Raoult *et al*. identified *R*. *prowazekii* in lice collected from Burundi jails, an observation that predicted the huge outbreak of epidemic typhus which erupted in refugee camps in Burundi two years later, in 1997 [1,32,33].

The present work contributes towards this approach, by studying the bacterial pathogens associated with head lice collected in two rural villages in Mali, where a high frequency of head lice infestation had previously been reported [34].

## Materials and methods

### Study area, sampling and ethics statement

The study was performed in January 2013 in two rural Malian villages, Donéguébougou (12°48’85”N 7°58’22”W) and Zorocoro (12°44’75”N 80°04’50”W), situated in close proximity in the Koulikoro region in a savanna zone. Lice were collected from patients presenting at the health centers in these two villages. All sampled individuals were thoroughly examined for the presence of both head and body lice. All visible head lice were removed from the hair using a fine-toothed comb. In total, 259 head lice samples were isolated from 56 individuals in the village of Donéguébougou and 341 head lice were isolated from 61 individuals in the village of Zorocoro. No body lice were found during the examination. General sanitary and hygienic conditions were poor. All the lice were preserved dry in sterile conditions at room temperature and sent to our laboratory in Marseille (France).

This study was approved by the Institutional Ethics Committee of the Faculty of Medicine of Pharmacy and Odontostomatology (permit no. 2013/113/CE/FMPOS). Written informed consent was obtained from the individuals involved or from their legal representatives in the case of children. The representatives of a local health center and the village elders accompanied the researchers for the duration of the study.

### DNA preparation

Prior to DNA isolation and in order to avoid external contamination, the surface of each louse was decontaminated as described previously [18], then each specimen was cut longitudinally into halves. One half was placed in a sterile tube and frozen for later use. The other was crushed in sterile Eppendorf tube and total-DNA was extracted using a DNA extraction kit, QIAamp Tissue Kit (Qiagen, Courtaboeuf, France) using the EZ1 apparatus following the manufacturer’s protocols and stored at 4°C until use in PCR amplifications.

### Molecular detection of the presence of pathogen DNA

#### Screening of pathogen DNA by qPCR

All DNA samples were screened using quantitative real-time PCR (qPCR) using previously reported primers and probes targeting the 16S rRNA gene of *Borrelia* spp. [35], the 23S gene of *Anaplasmataceae* spp. [36], the *gltA* gene of *Rickettsia* spp.[37], the *ompB* gene of *R*. *prowazekii* [38], the *pla* gene of *Yersinia pestis* [38], the *yopP* gene of *B*. *quintana* [25] and the IS1111 of *C*. *burnetii* [39]. In addition, all *B*. *quintana* and *C*. *burnetii* positive samples were confirmed by a second specific qPCR process targeting the *fabF3* gene and IS30A spacers respectively [25,39]. All the sequences of primers and probes used for qPCRs and conventional PCRs in this study are given in the supplementary files (S1 Table).

All qPCRs were performed using a CFX96^™^ Real-Time system (Bio-Rad Laboratories, Foster City, CA, USA). The final reaction volume of 20 μl contained 5 μl of the DNA template, 10 μl of Eurogentec^™^ Probe PCR Master Mix (Eurogentec, Liège, Belgium), 0.5 μM of each primer and 0.5 μM of the FAM-labeled probe. The thermal cycling conditions included one incubation step at 50°C for two minutes and an initial denaturation step at 95°C for three minutes, followed by 40 cycles of denaturation at 95°C for 15 seconds and annealing extension at 60°C for 30 seconds.

We included the DNA of each target bacteria as positive controls and master mixtures as negative controls to validate each PCR run. No amplifications were detected among the negative controls throughout the study. We considered samples to be positive when the cycle threshold (Ct) was lower than 35 Ct [40].

#### Conventional PCR and sequencing

All samples that tested positive using *Rickettsia* genus-specific primers were subjected to standard PCR targeting a 1177-bps fragment of *gltA* gene [41]. For the identification of *Anaplasmataceae* species, all positive samples were tested in two PCRs using a set of *Anaplasma* genus-specific primers targeting the 525-bps fragment of the *rpoB* gene and a *Ehrlichia* genus-specific set of primers targeting the 590-bps portion of the *groEL* gene (heat shock protein gene) [36]. In addition, we determined the multi-spacer typing (MST) of *C*. *burnetii* positive samples by amplifying three intergenic spacers (Cox2, Cox5 and Cox18) [42].

All PCR amplification was performed using a Peltier PTC-200 model thermal cycler (MJ Research Inc., Watertown, MA, USA). Reactions were carried out using the Hotstar Taq-polymerase (Qiagen), in accordance with the manufacturer’s instructions. Negative and positive controls were included in each assay. The success of amplification was confirmed by electrophoresis on a 1.5% agarose gel.

Purification of PCR products was performed using NucleoFast 96 PCR plates (Macherey-Nagel EURL, Hoerdt, France) as per the manufacturer’s instructions. The amplicons were sequenced using the Big Dye Terminator Cycle Sequencing Kit (Perkin Elmer Applied Biosystems, Foster City, CA) with an ABI automated sequencer (Applied Biosystems). The electropherograms which were obtained were assembled and edited using ChromasPro software (ChromasPro 1.7, Technelysium Pty Ltd., Tewantin, Australia) and compared with those available in the GenBank database by NCBI BLAST (http://blast.ncbi.nlm.nih.gov/Blast.cgi).

### Mitochondrial clade of lice

#### Determination of louse mitochondrial clade by qPCR assays

To determine the mitochondrial clades of the lice studied, all the DNA samples were analyzed using clade-specific qPCR assays that targeted a portion of the cytochrome b (*cytb*) gene, specific to clades A, D, B and C described in our previous study [12]. It is important to note that when we performed the design of the qPCR specific to clade C, clade E was classified as a sub-clade within clade C [12], therefore, this qPCR detected both clades C and E. To discriminate between them, we performed another qPCR essay specific only to clade E, targeting 129-bp of *cytb* (nucleotide position 605–734 of *cytb* gene). The design was performed and optimized for specificity and sensitivity as described previously[12]. Subsequently, all the lice qPCR which were clade C+E positive were further subjected to qPCR which were clade E specific. We used lice with previously identified clades as positive controls. Negative controls were included in each assay.

#### Cytochrome b amplification and sequencing

For phylogenetic study, forty-five head lice of the total collected were randomly selected and subjected to standard PCR targeting a 347-bp fragment of the *cytb* gene using the primers and conditions as previously described [8]. Successful amplification was confirmed via gel electrophoresis and amplicons were prepared and sequenced using similar methods as described above for bacteria.

### Testing of blood meals in head lice

For blood meal identification, only head lice specimens with positive bacterial-DNA results were assayed by PCR using the vertebrate-universal specific primers 16SA and 16SB (Table 1) to amplify a 580-bps fragment of the vertebrate host mitochondrial 16S ribosomal RNA as described previously [43]. Successful amplification was confirmed via gel electrophoresis and amplicons were prepared and sequenced using similar methods as described above for bacteria.

**Table 1:** Haplotype frequency of Mali head lice identified per village.

Haplotype	Donéguébougou	Zorocoro	*Total*	Acc. no.
E39	5	1	6	KM579560
E46	3	9	12	KX249780
E47	6	2	8	KX249781
**E48**	5	10	15	**KY937987**
**E49**	1	1	2	**KY937988**
**E50**	0	1	1	**KY937989**
**E51**	0	1	1	**KY937990**
***Total***	20	25	45	

### Data analysis

For the head lice *cytb* sequences obtained in this study, unique haplotypes were defined using DnaSPv5.10 and compared with all the reference *cytb* haplotypes as described previously [12]. All obtained sequences of *Rickettsia* and Anaplasmataceae species were analyzed using BLAST (www.ncbi.nlm.nih.gov/blast/Blast.cgi) and compared to sequences in the GenBank database. For *C*. *burnetii*, all the sequences obtained from the three spacers were compared with those reported in the reference database available on the website (http://ifr48.timone.univ-mrs.fr/MST_Coxiella/mst). Sequences of three spacers from all available genotypes were concatenated and aligned using CLUSTAL W for multisequence alignment implemented in MEGA software version 6.06[44].

A maximum-likelihood method was used to infer the phylogenetic analyses and tree reconstruction was performed using MEGA software version 6.06 [44].

## Results

### Lice clade and phylogenetic analysis

In total, 600 head lice were collected from 117 individuals living in two villages in Mali and all were tested by qPCRs to determine their clade. Our results show that all the head lice tested (600/600; 100%) belonged to clade E.

**Fig 1:**
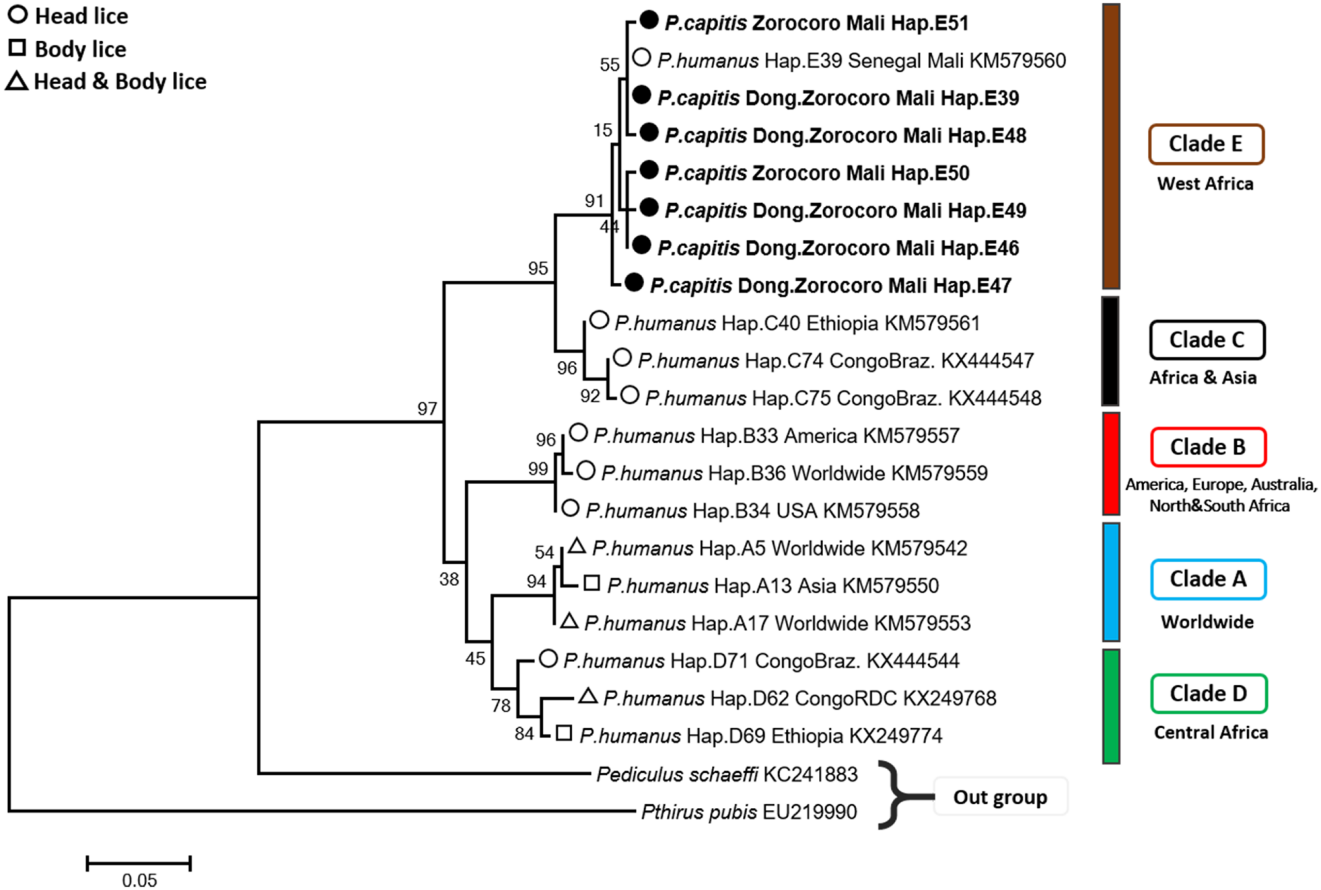
Phylogenetic tree showing the relationship between haplotypes identified in this study with other *Pediculus humanus* haplotypes. The *cytb* sequences were aligned using CLUSTALW, and phylogenetic inferences were conducted in MEGA 6 using the maximum likelihood method based on the Kimura 2-parameter for nucleotide sequences. The GenBank accession numbers are indicated at the end. Statistical support for the internal branches of the trees was evaluated by bootstrapping with 1,000 iterations. The codon positions included were 1st+2nd+3rd+Noncoding. There was a total of 270 positions in the final dataset. The scale bar represents a 1% nucleotide sequence divergence.

For phylogenetic study, a total of 45 head lice *cytb* sequences were analyzed, defining seven different haplotypes, of which four are novel, referred to here E48, E49, E50 and E51, while the remaining three haplotypes possessed the E39 (previously referred as C39) haplotype from Mali and Senegal, and E46 and E47 (previously referred as C46 and C47) haplotypes from Mali. Haplotype E48 was the most prevalent (33.3%), followed by haplotype E39 (26.6%). All the identified haplotypes, together with references from the body and head lice haplogroups were used to construct a maximum-likelihood (ML) tree (Fig 1). All the Malian head lice *cytb* sequences were clustered with clade E from West Africa (Senegal and Mali). The novel haplotype sequences identified have been deposited in GenBank (Table 1).

### Molecular detection of bacterial pathogens

In this study, the qPCR investigation of all 600 head lice samples for *Borrelia* spp., *R*. *prowazekii* and *Y*. *pestis* produced no positive results. However, we obtained positive results when testing for the presence of *B*. *quintana*, *C*. *burnetii*, *Rickettsia* spp. and *Anaplasmataceae* species.

The DNA of *B*. *quintana* was detected in three of 600 (0.5%) head lice collected from two of 117 (1.7%) persons. All the infected lice were from the village of Donéguébougou. No positive samples were found in the village of Zorocoro.

**Fig 2:**
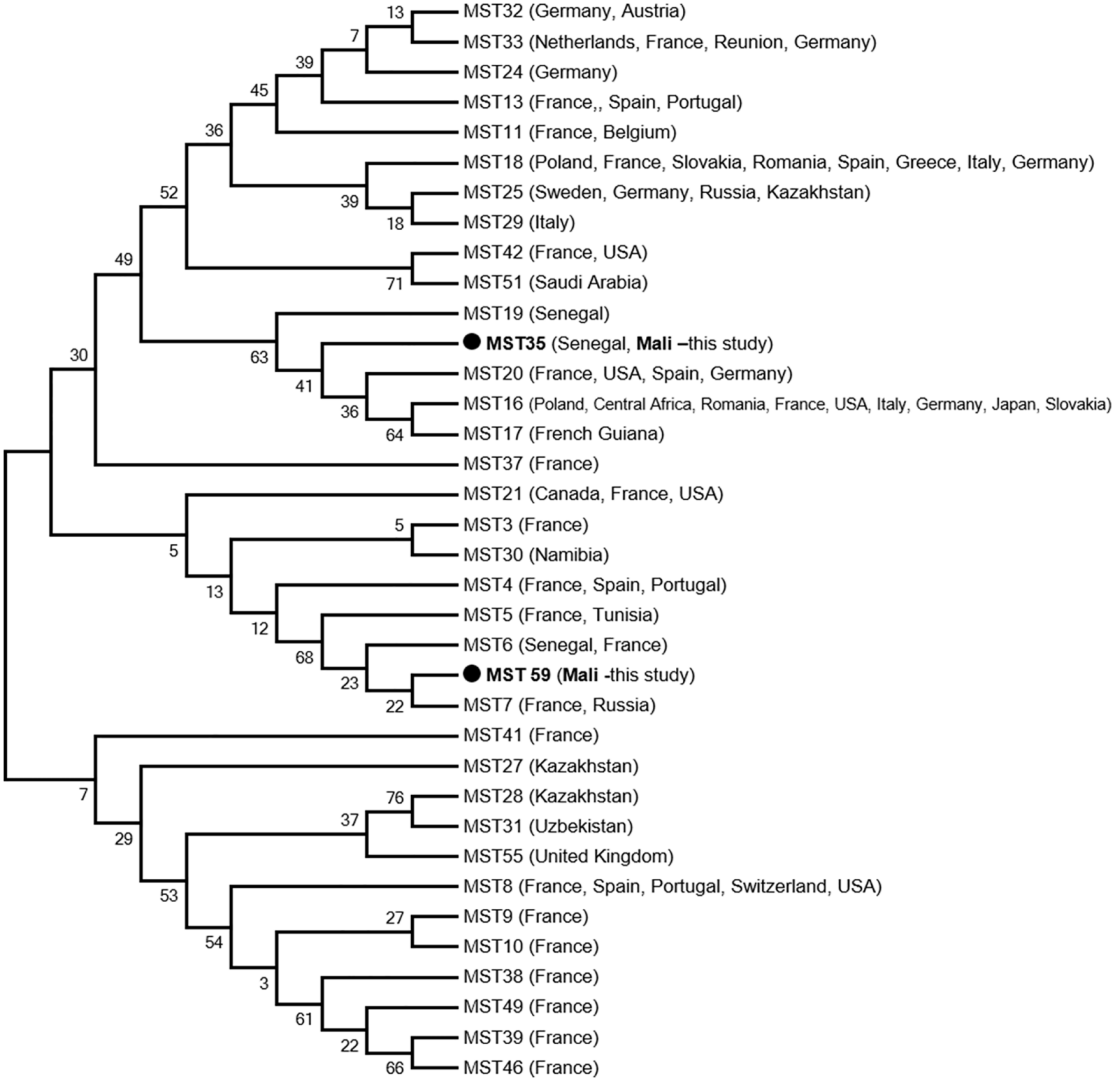
Phylogenetic position of identified genotypes of *C*. *burnetii*, the agent of Q fever. The concerned sequences (COX2, 5 and 18) were aligned using CLUSTALW, and phylogenetic inferences was conducted in MEGA 6 using the maximum likelihood method, with the complete deletion option, based on the Kimura 2-parameter for nucleotide sequences. There was a total of 1,247 positions in the final dataset.

Seven of 600 (1.16%) lice samples collected from six of 117 (5.1%) persons tested positive by qPCR using both systems for the presence of *C*. *burnetii* DNA. Four of the seven (57.14%) positive lice were from Zorocoro and the remaining three (42.85%) positive lice were from Donéguébougou. We performed MST genotyping of *C*. *burnetii* positive lice. Genotype 35, previously recorded in Senegal, was found in one louse from Zorocoro. Another new genotype (genotype 59) was found in two lice from Donéguébougou. The phylogenetic position of these genotypes is shown in Fig 2. Interestingly, these two lice were collected from the same person. All attempts to genotype the other positive samples were unsuccessful, possibly because of low DNA concentration.

**Fig 3:**
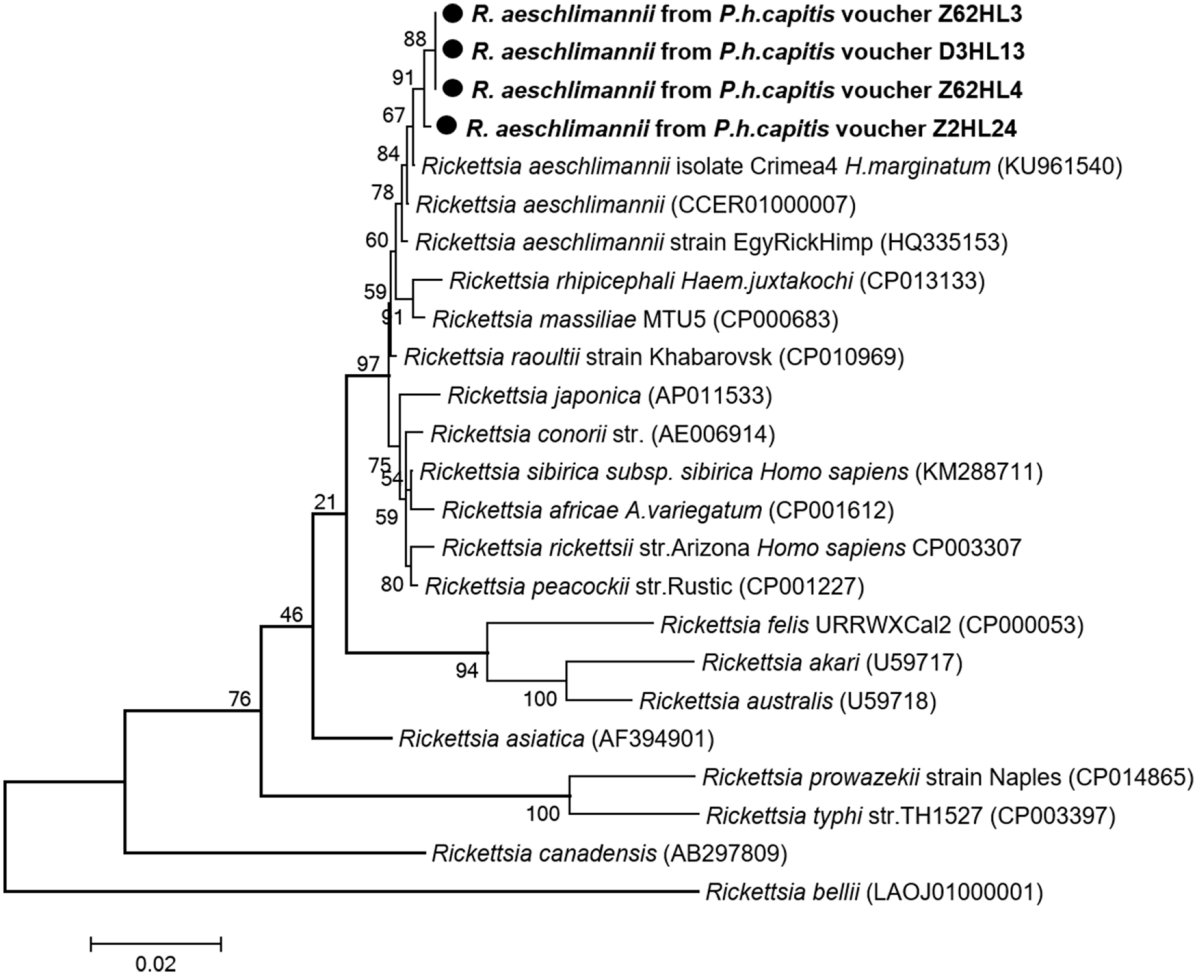
Phylogenetic tree highlighting the position of *Rickettsia* spp. identified in the present study compared to other *Rickettsia* bacteria available on GenBank. The *gltA* sequences were aligned using CLUSTALW, and phylogenetic inferences were conducted in MEGA 6 using the maximum likelihood method, with the complete deletion option, based on the Kimura 3-parameter for nucleotide sequences. The GenBank accession numbers are indicated at the end. Statistical support for the internal branches of the trees was evaluated by bootstrapping with 1,000 iterations. The codon positions included were 1st+2nd+3rd+Noncoding. There was a total of 1,161 positions in the final dataset. The scale bar represents a 2% nucleotide sequence divergence.

*Rickettsial DNA* was detected by qPCR targeting the *gltA* gene in four of 600 (0.6%) head lice collected from three of 117 (2.56%) persons (Table 2). All positive samples were also amplified by conventional PCR using primers targeting a 1,158-bps fragment of the same gene. Three of the four obtained sequences (sample vouchers: Z62HL3, Z62HL4 and D3HL13) were 100% identical to one another, differing by one nucleotide base from the fourth sequence (sample voucher: Z2HL24) and were identified as *R*. *aeschlimannii* based on a BLAST search, sharing 99, 65% (1,154 of 1,158 base positions in common) and 99, 74% (1,155 of 1,158 base positions in common) similarity with a reference strain of *R*. *aeschlimannii* isolate Crimea-4 (GenBank number KU961540), respectively. These results were also confirmed by a specific qPCR for *R*. *aeschlimannii* [39]. The phylogenetic position of this *Rickettsia* is given in Fig 3. The partial nucleotide sequence of the *gltA* gene obtained in this study was deposited in the GenBank under accession number: KY937991- KY937992.

**Table 2:** Summary of the pathogens detected in head lice collected from infested individuals in two rural villages in Mali, 2013.

	Donéguébougou		Zorocoro		*Total*	
Bacterial species	Persons N = 56	Head lice N = 259	Persons N = 61	Head lice N = 341	Persons N = 117	Head lice N = 600
***B*. *quintana***	2	3	0	0	2 (1.7%)	3 (0.5%)
***C*. *burnetii***	3	3	3	4	6 (5.1%)	7 (1.16%)
***R*. *aeschlimannii***	1	1	2	3	3 (2.56%)	4 (0.6%)
***Ehrlichia***	6	9	4	5	10 (8.54%)	14 (2.3%)
***Anaplasma***	2	2	0	0	2 (1.7%)	2 (0.3%)
*Borrelia*	0	0	0	0	0	0
*Y*. *pestis*	0	0	0	0	0	0
*R*. *prowazekii*	0	0	0	0	0	0

**Fig 4:**
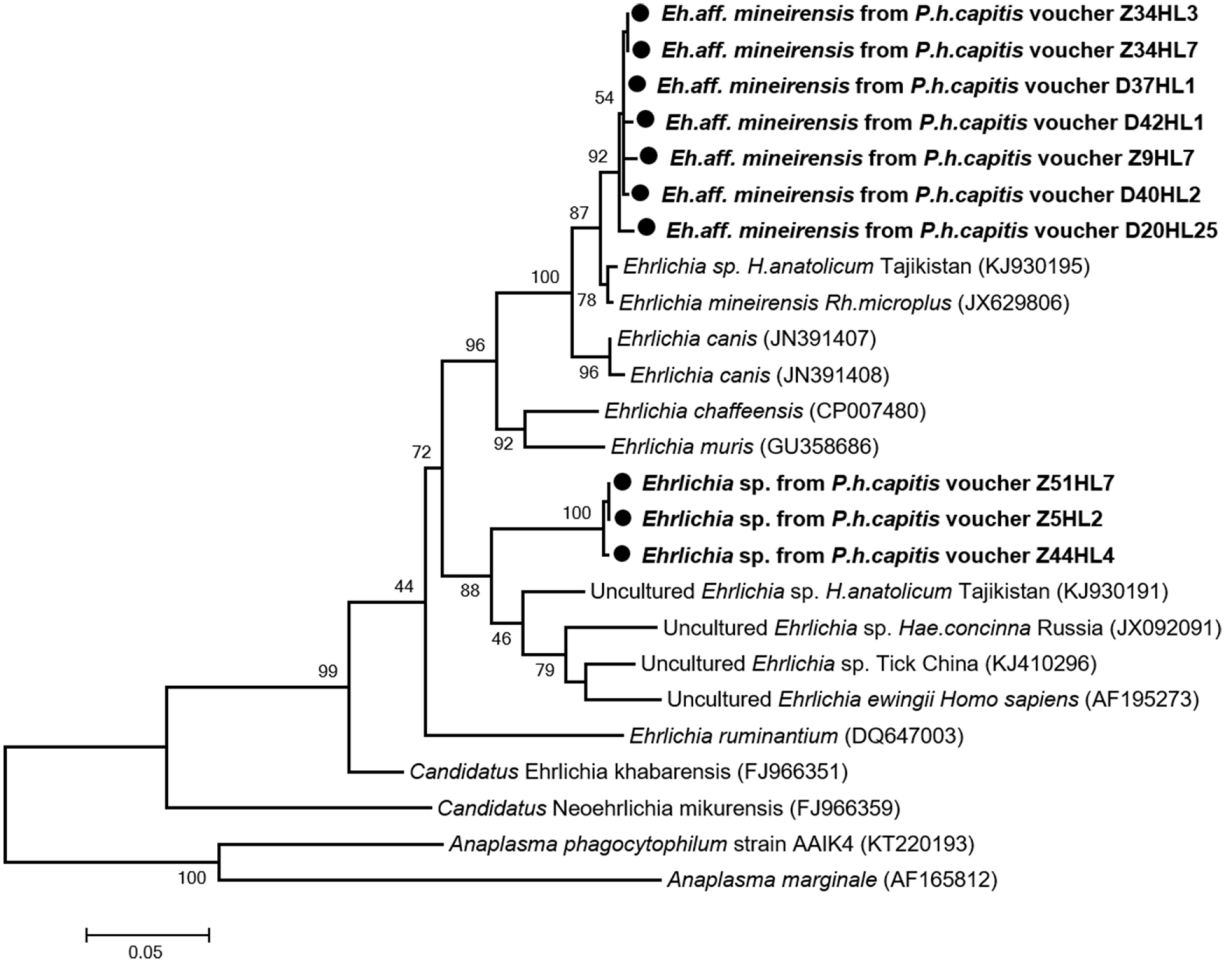
Phylogenetic tree highlighting the position of *Ehrlichia* spp. identified in the present study compared to other *Ehrlichia* bacteria available on GenBank. The *groEl* sequences were aligned using CLUSTALW, and phylogenetic inferences was conducted in MEGA 6 using the maximum likelihood method based on the Kimura 3-parameter model for nucleotide sequences. The GenBank accession numbers are indicated at the end. Statistical support for the internal branches of the trees was evaluated by bootstrapping with 1,000 iterations. The codon positions included were 1st+2nd+3rd+Noncoding. There was a total of 570 positions in the final dataset. The scale bar represents a 5% nucleotide sequence divergence.

For *Anaplasmataceae*, the 23S-based qPCR screening showed 15 out of 600 (2.5%) head lice, collected from 11 of 117 (9.4%) individuals, contained DNA of the *Anaplasmataceae* species. Conventional PCR and sequencing using specific *Ehrlichia* genus-primers targeting a 590-bps fragment of g*roEL* gene showed that 14 of the 15 lice tested were positive for *Ehrlichia*. Comparison with the GenBank database sequences showed that 11/14 of these sequences form new genotypes closely related to not officially recognized species *E*. *mineirensis* UFMG-EV (GenBank number JX629806) with 98.26–98.6% similarities. These new genotypes, referred to here as *E*. *aff*. *mineirensis*, together with *E*. *mineirensis* are clustered within the clade of *E*. *canis*, as shown in the phylogenetic tree (Fig 4). For three of the 14 remaining sequences, BLAST analysis showed a homology score of under 93% which means that these sequences are likely to be a potential undescribed new species. The closest officially recognized species is *E*. *ewingii* (GenBank number AF195273) with 90.4% identity. These three sequences have one to two SNP between them. In the phylogenetic tree (Fig 4), the sequences of this potential new *Ehrlichia* sp., provisionally referred to here as “Mali” form a separate and well-supported (bootstrap value 88) branch, which clustered together within the clade that contains *E*. *ewingii* from human and other uncultured *Ehrlichia* sp. from hard ticks.

**Fig 5:**
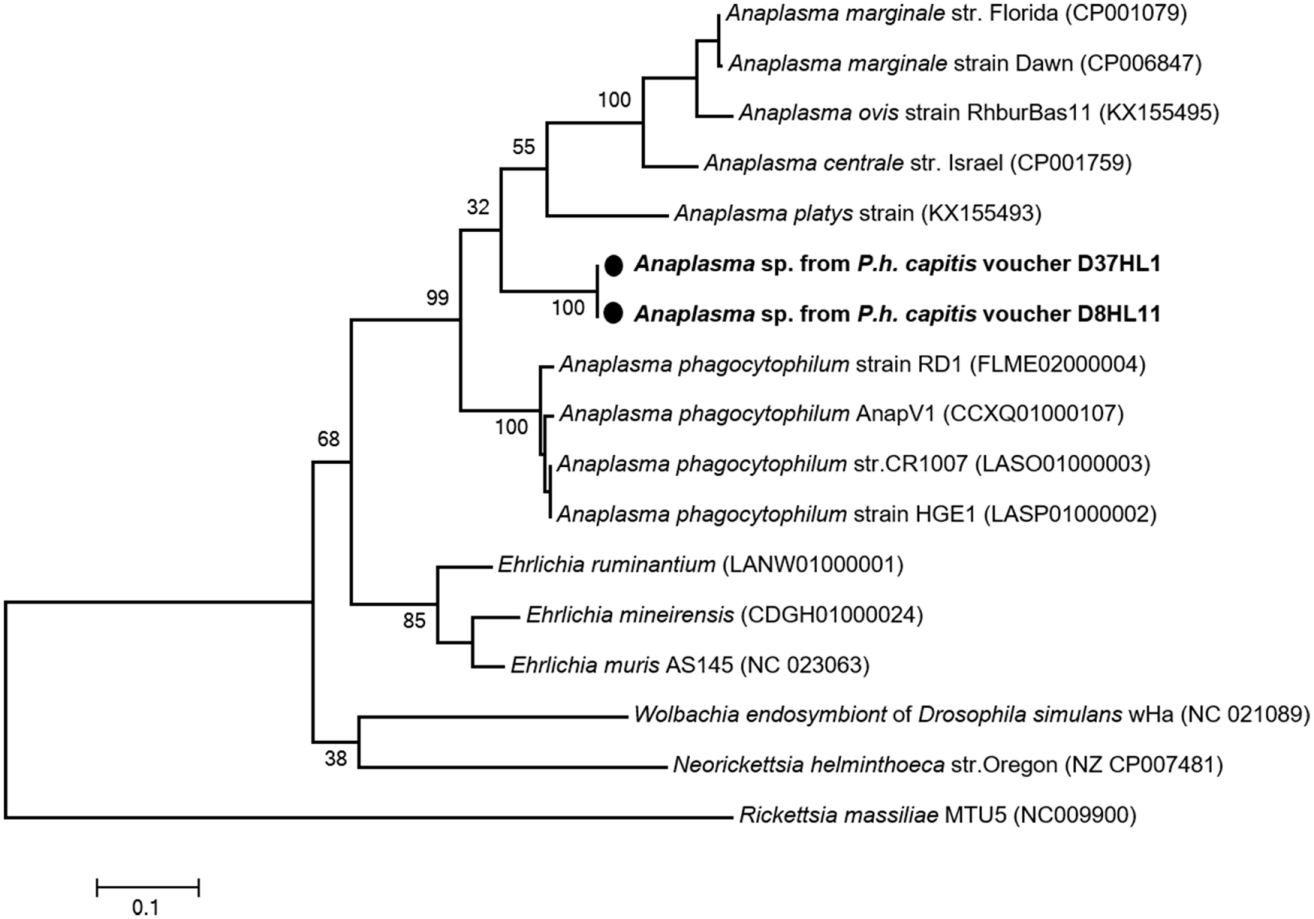
Phylogenetic tree highlighting the position of *Anaplasma* spp. identified in the present study compared to other Ehrlichia bacteria available on GenBank. The *rpoB* sequences were aligned using CLUSTALW, and phylogenetic inferences were conducted in MEGA 6 using the maximum likelihood method based on the Kimura 3-parameter for nucleotide sequences. The GenBank accession numbers are indicated at the end. Statistical support for internal branches of the trees was evaluated by bootstrapping with 1,000 iterations. The codon positions included were 1st+2nd+3rd+Noncoding. There was a total of 429 positions in the final dataset. The scale bar represents a 10% nucleotide sequence divergence.

Using the specific *Anaplasma* genus-primers targeting 525-bps fragment of the *rpoB* gene, we found that two of the 15 lice tested, collected from two individuals, were positive for *Anaplasma* spp. Interestingly, one of the positive lice was also co-infected with *E*. *aff*. *mineirensis*. All the infested lice were from Donéguébougou. No positive sampled were found in Zorocoro. A BLAST search showed that these sequences probably belong to an undescribed species, *Anaplasma* sp., provisionally referred to here as “Mali”, because only 83% (327/395-bps), 81% (317/392-bps), 80% (316/394-bps) and 80% (315/392-bps) similarities were observed, respectively, with the *rpoB* gene of *A*. *phagocytophilum* (GenBank number FLME02000004), *A*. *centrale* (GenBank number CP001759), *A*. *marginale* (GenBank number CP001079) and *A*. *platys* (GenBank number KX155493). The phylogenetic position of these *Anaplasma* are given in Fig 5.

Accordingly, *E*. *aff*. *mineirensis* showed an infection rate of 1.8% (11/600) of the total number of lice tested, *Ehrlichia* sp. “Mali” showed an infection rate of 0.5% (3/600) lice tested and *Anaplasma sp*. “Mali” showed an infection rate of 0.5% (2/600) lice tested, including one co-infection *E*. *aff*. *mineirensis/Anaplasma* sp. “Mali”. The partial nucleotide sequence of the *groEl* gene of *Ehrlichia* and *rpoB* gene of *Anaplasma* obtained in this study were deposited in the GenBank under the accession numbers KY937978- KY937986.

### Blood meal identification in head lice

We also performed blood meal analysis in the 29 head lice specimens which were positive for at least one pathogen tested. As expected, DNA from human blood was detected in all lice tested. Thus, 25 of the 29 obtained sequences showed 100% identity, while the remaining four sequences showed 99.83–99.65% similarities with the 16S ribosomal RNA of *Homo sapiens* mitochondrial sequences available in the Genbank database.

## Discussion

Human lice infestation remains prevalent worldwide. Surprising and novel insights into the evolution of these ancient and highly intimate scourges of the human race, their bacterial disease agents, and the epidemiology of louse-borne diseases are stimulating a renewal of interest in these bloodsucking insects.

Here we provide results of head lice screening from two rural villages of Mali in the savanna zone, where a high rate infestation had previously been reported in 88% of the 112 individuals studied, reflecting the low socioeconomic level in this area [34]. During an epidemiological investigation in New York, the distribution of head lice was associated with gender (boys’ heads are usually shaved), age, socioeconomic status, crowding, methods of storing garments, and family size [5]. Poverty and ignorance appeared to contribute to the persistence of the disease [5]. The mtDNA analysis of the 600 head lice collected from 117 Malian individuals, showed that all the head lice tested belonged to clade E, specific to West Africa, as reported by others [6,34].

*B*. *quintana*, the causative agent of trench fever, has a long history of association with humans dating back over 4,000 years [45]. Infection was common in France in the 18^th^ century, during Napoleon’s Russian war, and during World Wars I and II [1,5,46]. It is currently regarded as a re-emerging pathogen in poor countries, as well as in developed countries among the homeless population, where it is responsible for a range of clinical manifestations in humans, including asymptomatic chronic bacteremia, endocarditis and bacillary angiomatosis [1,5]. It is a very common cause of endocarditis in North Africa [47,48].

For a long time, it was thought that *B*. *quintana* was only transmitted by body lice in humans. It was, moreover, found in cats [49] and some human cases have been linked to contact with kittens (S2 Table) [50]. Furthermore, in recent years, *B*. *quintana*-DNA has frequently been detected in head lice collected from impoverished populations such as the homeless or Nepalese children living in slums or on the streets, who are usually infested with both head and body lice [27,51,52], as well as in head lice and head louse nits without concurrent body lice infestation [14,26,28], highlighting the possible role of head lice as an additional vector in the transmission of *B*. *quintana* to humans (S2 Table). In this study, we found *B*. *quintana* DNA in three of the 600 head lice studied with no evidence of body lice. Our work reinforces findings from previous studies that head lice, as is the case of body lice, may act as vectors of *B*. *quintana*.

For the first time, the presence of *B*. *quintana* in Malian head lice has been shown. All the positive lice were collected from two people living in the same village, Donéguébougou. No positive samples were found in Zorocoro. The studies conducted in Mali by Sangaré *et al*. failed to detect this bacterium in head lice collected from another Malian village, Diankabou, situated in the Sahelian zone [6,34]. Of all these three villages studied, *B*. *quintana* was found in only one village, suggesting a local occurrence of this pathogens. All *B*. *quintana* positive head lice were clade E, the unique clade found in the studied era. In recent studies from neighboring Senegal, *B*. *quintana* was also detected in head lice clade C, which is now recognized as clade E, the same clade found in Mali, as well as in head lice belonging to clade A [14,28]. *B*. *quintana* has also been reported in head lice clades C and D from Ethiopia and the Democratic Republic of the Congo, respectively [10,29,53], suggesting that all clades of head lice, except clade B from which no infection has been reported to date, have the potential to serve as vectors for *B*. *quintana*. Given the scale of head lice infestation around the world, it is of paramount importance to address their competence as potential disease vectors.

In this study, we also assessed our collected lice for the presence of *C*. *burnetii*, *Rickettsia* spp and *Anaplasmataceae*. These bacteria are usually not associated with human lice, so we used additional tools to confirm that the amplified microorganisms were really associated with human lice. We amplified and sequenced louse *cytb* from each of positive samples and identified the human blood meal inside each arthropod, so we are sure that we amplified these bacteria from engorged human lice. Although all these pathogenic bacteria are not correlated with louse transmission, it is feasible that lice can transmit any agent of chronic bacteremia that is ingested with the blood meal and capable of surviving in the insect’s midgut [1]. Furthermore, lice have been demonstrated to be capable of mechanical transmission for virtually all microorganisms tested, including *C*. *burnetii* and *Rickettsia* species [1].

*C*. *burnetii*, the causative agent of Q fever, is a worldwide zoonotic disease. The bacterium has a wide host range, including wild and domestic mammals, birds, reptiles, and arthropods, mainly ticks [54]. Infection in humans, usually through aerosol inhalation, can be acute or chronic and the disease exhibits a wide spectrum of clinical manifestations [54,55]. Infections with *C*. *burnetii* has been reported throughout the African continent with a high prevalence in Senegal, indicating that Q fever should be considered as a significant public health threat in Africa [55,56]. In Mali, only two serological studies have thus far been performed on humans. The first study was conducted by Tissot-Dupont *et al*. (1995) and found a seroprevalence of 24% in healthy urban-dwelling people [57]. The second study was conducted by Steinmann *et al*. (2005) and showed that 40% of 156 mainly adult febrile patients had antibodies against *C*. *burnetii*, with 10% of positives having a serological profile suggesting acute infection [58]. Most recently, another study performed on 100 febrile Malian patients (in the village of Diankabou) based on qPCR showed no positive results [56]. One molecular study conducted on both head and body lice from Ethiopia showed no evidence of *C*. *burnetii* in all the 98 louse pools tested [53]. The findings from our study showed that 1% of 600 head lice tested infesting 5% of 117 persons studied were *C*. *burnetii* DNA positive. Four of the seven positive lice were from Zorocoro and the remaining three positive lice were from Donéguébougou. To the best of our knowledge, this is the first molecular evidence of the presence of *C*. *burnetii* DNA in head lice infesting individuals from Mali. Although human lice are not known vectors of *C*. *burnetii*, it has been shown that, under experimental conditions, it is possible to infect body lice with *C*. *burnetii* [59]. There is also a field observation that lice collected in a place where an epidemic of Q fever occurred three months previously are capable of transmitting *C*. *burnetii* to guinea pigs [20,59]. Our results from Mali, together with data from the literature, suggest that the role of human lice in the epidemiology of Q fever should be investigated further.

MST genotyping showed the presence of genotype 35 in one louse from Zorocoro. This genotype was also detected previously in West Africa, in febrile patients and ticks from Senegal [55,56]. Another new genotype (genotype 59) was found in two lice collected from the same person in Donéguébougou. A phylogenetic tree based on concatenated sequences (Fig 2) shows that this newly found genotype is mostly related to MST genotypes 6 and 7. As reported in the reference database (http://ifr48.timone.univmrs.fr/mst/coxiella_burnetii/strains.html), genotype 7 was detected in human blood from France and Russia, and genotype 6 was detected in ticks from Senegal and clinical human samples (heart valve and human sera) from France, with no available epidemiological data.

Rickettsial species are transmitted by hematophagous arthropods, which contain several agents of human disease [60]. *R*. *prowazekii*, a member of the typhus group, is the only known species naturally associated with human lice, in which the body louse is the natural vector and the head louse has been proposed as an additional vector, demonstrated only under laboratory conditions [1,24]. We didn't find this pathogen in the lice we studied. Although the human louse is not a known vector of rickettsiae species belonging to the spotted fevers group (SFGR), an experimental infection demonstrated that the body louse was able to acquire, maintain, and transmit both *R*. *rickettsii* and *R*. *conorii*, suggesting that it may play a role, under favorable epidemiologic circumstances, in their transmission to humans [22]. In this study, we demonstrate for the first time the presence of *R*. *aeschlimannii*-DNA, another member of SFG, in 0.6% of the 600 head lice collected from 2.56% of 117 Malian individuals.

*R*. *aeschlimannii* causing spotted fever was first identified in a patient returning from Morocco [60]. In West Africa, including Mali, this rickettsia was mainly detected in *Hyalomma* ticks which appear to be the main vectors and reservoirs [60]. Until now, no human cases of *R*. *aeschlimannii*-associated spotted fever has been reported from these countries [60]. Our findings show additional evidence of the presence of the species in Mali being detected in human head lice.

Within the *Anaplasmataceae* family, two significant genera *Anaplasma* and *Ehrlichia*, are worldwide tick-borne pathogens that can cause serious illness in a variety of hosts, including humans [61]. These pathogens are not frequently reported in West Africa, with most reports concerning veterinary pathogens in tick vectors [62]. To date, no human cases of anaplasmosis have been reported in Mali. In 1992, a serological survey against *E*. *chaffeensis* (the agent of human monocytic ehrlichiosis) in human sera from eight African countries, including Mali, indicated that human ehrlichioses might occur on the continent [63] and a case (diagnosed by serology only) was subsequently reported from Mali [64]. *E*. *chaffeensis* has also been identified by PCR in 10% of febrile patients in Central Africa [65], and *E*. *ruminantium*-like organisms have been implicated in human infections in South Africa [66]. In the present study, the DNA of *Anaplasmataceae* was detected in 2.5% of 600 head lice, collected from 9.4% of 117 individuals. To the best of our knowledge, this is the first evidence of the presence of *Anaplasmataceae* DNA in human head lice.

*Ehrlichia* was detected in 14 of 600 (2.3%) head lice collected from 10/117 (8.54%) individuals. Specifically, three of 14 *Ehrlichia* sequences form a potentially undescribed new species, clustered together within the clade containing *E*. *ewingii* from human and other uncultured *Ehrlichia* sp. from hard ticks. The remaining 11 sequences form new genotype closely related to the not officially recognized species *E*. *mineirensis*, a new emerging clade of cattle *Ehrlichia* pathogens within the *E*. *canis* group, the etiologic agent of canine monocytic ehrlichiosis. Recent reports suggested that this species might also be a human pathogen [61]. In 2001, two new ehrlichial genotypes of the *E*. *canis* group (which includes *E*. *chaffeensis*, *E*. *ruminantium*, *E*. *muris* and *E*. *ewingii*) were reported in *Rhipicephalus muhsamae* from Mali and in *Hyalomma truncatum* from Niger [67]. Because in this group, as within each group of ehrlichiae, members share homologous surface antigens and thus cross-react extensively in serologic assays, the authors suggested that these two genotypes may also be organisms responsible for serologic cross-reactions, including in serosurveys and case reports of human ehrlichioses in Africa for currently recognized human pathogenic ehrlichiae [67].

Finally, the DNA of a potential new *Anaplasma* species was detected in two of 600 (1.58%) head lice collected from in two persons. one of the positive lice was also co-infected with *E*. *aff*. *mineirensis*. Blast analysis of the *rpoB* gene showed that this *Anaplasma* sp. was significantly different from all other previously reported *Anaplasma* species. The closest related species was, with 83% similarities, *A*. *phagocytophilum*, the causative agent of human granulocytic anaplasmosis. This species was recently reported in neighboring Senegal [68].

However, the detection of these potential new species has its limitations, as not all previously described species of *Ehrlichia* and *Anaplasma* are already molecularly characterized, so the detection of a ‘new’ genotype may, in fact, be the re-discovery of an old, incompletely characterized species [62]. Further studies are required to clarify whether these new genetic variants represent a new species.

Furthermore, molecular evidence for the presence of the DNA of these bacteria in head lice cannot distinguish between transient infections, pathogens accidentally acquired from the blood of infected individuals, and those established in a competent vector which can maintain and transmit the pathogen. Nevertheless, all pathogenic species from *Anaplasma* and *Ehrlichia* genus are known to be obligatory transmitted by arthropods. Further studies are needed to determine whether the head louse can act as a vector of these bacteria species.

## Conclusions

In conclusion, our finding of several Malian head lice which were positive for *B*. *quintana*, *C*. *burnetii*, *R*. *aeschlimannii*, *Anaplasma* and *Ehrlichia* is alarming. Currently, our understanding of the role of human head lice in the epidemiology of these emerging pathogenic bacteria is limited. Since head lice feed only on human blood [5,32], an assumption which is further evidenced in our study by the identification of human blood meal in all head lice with positive bacterial-DNA, the obtained results imply that the acquired infections are from the blood of patients with ongoing bacteremia. Hence, our study provides a starting point for epidemiological studies in this area and active survey programs should be encouraged.

In Mali, as in the case of several poor African countries, the laboratory capacity to diagnose these infections is often lacking, while many potential emerging pathogens of concern might already be infecting humans but have not yet been detected through disease surveillance. The durability of lice as a sample and the ease with which they can be collected and transported to reference laboratories where suitable molecular biological approaches are available, enhance their potential use as an efficient epidemiological witness for the monitoring and surveillance of emerging pathogens circulating in humans that is critical for the prediction of future disease outbreaks and epidemics at an early stage.

## Supporting information

S1 Table Oligonucleotide sequences of primers and probes used for real-time PCRs and conventional PCRs in this study. (DOCX)

S2 Table Source of *B*. *quintana* infection in humans. (RTF)
