# Supplementary material for: Detection of bacterial pathogens including potential new species in human head lice from Mali
Source: PLoS One. 2017 Sep 20;12(9):e0184621. doi: 10.1371/journal.pone.0184621 (PMC5606924; doi:10.1371/journal.pone.0184621)
Supplement: S1 Table — (DOCX) [file pone.0184621.s001.docx]

**S1 Table. Oligonucleotide sequences of primers and probes used for quantitative real-time PCRs and conventional PCRs in this study.**

| **Target** | **Name** | **Primers (5’-3’) and probes** | **Source** |
| --- | --- | --- | --- |
| ***Pediculus humanus***  *Cytochrome b* | Duplex A-D | F_ GATGTAAATAGAGGGTGGTT | [12] |
|  |  | R_ GAAATTCCTGAAAATCAAAC |  |
|  |  | FAM-CATTCTTGTCTACGTTCATATTTGG-TAMRA |  |
|  |  | VIC-TATTCTTGTCTACGTTCATGTTTGA-TAMRA |  |
|  | Duplex B-C/E | F_ TTAGAGCGMTTRTTTACCC | [12] |
|  |  | R_ AYAAACACACAAAAMCTCCT |  |
|  |  | FAM-GAGCTGGATAGTGATAAGGTTTAT-MGB |  |
|  |  | VIC-CTTGCCGTTTATTTTGTTGGGGTTT-TAMRA |  |
|  | Monoplex E | GGT TGG AAT TGG ATA GTG AT | This study |
|  |  | GGG TCC ATA AAG AAA TCC G |  |
|  |  | FAM- TAG GAG GCT TTG TGT GTC TAT CCT -TAMRA |  |
|  | *Cytb* | F_GAGCGACTGTAATTACTAATC | [8] |
|  |  | R_CAACAAAATTATCCGGGTCC |  |
| ***Rickettsia* spp**  citrate synthase (*gltA*) | RKNDO3 | F_GTGAATGAAAGATTACACTATTTAT | [37] |
|  |  | R_GTATCTTAGCAATCATTCTAATAGC |  |
|  |  | FAM-CTATTATGCTTGCGGCTGTCGGTTC-TAMRA |  |
|  | *gltA* | F_ATGACCAATGAAAATAATAAT | [40] |
|  |  | R_CTTATACTCTCTATGTACA |  |
| ***Rickettsia prowazekii***  *rOmpB* gene | *ompB* | F_AATGCTCTTGCAGCTGGTTCT | [37] |
|  |  | R_TCGAGTGCTAATATTTTTGAAGCA |  |
|  |  | FAM-CGGTGGTGTTAATGCTGCGTTACAACA-TAMRA |  |
| ***Yersinia pestis***  plasminogen activator gene | PLA | F_ATG GAG CTT ATA CCG GAA AC | [38] |
|  |  | R_GCG ATA CTG GCC TGC AAG |  |
|  |  | FAM-TCCCGAAAGGAGTGCGGGTAATAGG-TAMRA |  |
| ***Borrelia* spp**  *16S ribosomal RNA* | Bor16S | F_AGCCTTTAAAGCTTCGCTTGTAG | [35] |
|  |  | R_GCCTCCCGTAGGAGTCTGG |  |
|  |  | FAM-CCGGCCTGAGAGGGTGAACGG-TAMRA |  |
| **Anaplasmataceae**  23S ribosomal RNA | TtAna | F_TGACAGCGTACCTTTTGCAT | [36] |
|  |  | R_GTAACAGGTTCGGTCCTCCA |  |
|  |  | FAM-GGATTAGACCCGAAACCAAG-TAMRA |  |
| ***Anaplasma* spp** *rpoB* gene | Ana-rpoB | F_GCTGTTCCTAGGCTYTCTTACGCGA | [36] |
|  |  | R_AATCRAGCCAVGAGCCCCTRTAWGG |  |
| ***Ehrlichia* spp**  *groEL* gene | Ehr-groEL | F_GTTGAAAARACTGATGGTATGCA | [36] |
|  |  | R_ACACGRTCTTTACGYTCYTTAAC |  |
| ***Bartonella* quintana**  Hypothetical intracellular effector | yopP | F_ TAAACCTCGGGGGAAGCAGA | [25] |
|  |  | R_ TTTCGTCCTCAACCCCATCA |  |
|  |  | FAM-CGTTGCCGACAAGACGTCCTTG-TAMRA |  |
| 3-oxoacyl-synthase gene | fabF3 | F_ GCGGCCTTGCTCTTGATGA | [25] |
|  |  | R_ GCTACTCTGCGTGCCTTGGA |  |
|  |  | FAM-TGCA GCAGGTGGAGAGAACGTG-TAMRA |  |
| ***Coxiella burnetii***  Spacer IS1111 | IS1111 | F_CAAGAAACGTATCGCTGTGGC | [39] |
|  |  | R_CACAGAGCCACCGTATGAATC |  |
|  |  | FAM-CCGAGTTCGAAACAATGAGGGCTG-TAMRA |  |
| Spacer IS30A | IS30A | F_CGCTGACCTACAGAAATATGTCC | [39] |
|  |  | R_GGGGTAAGTAAATAATACCTTCTGG |  |
|  |  | FAM-CATGAAGCGATTTATCAATACGTGTATGC-TAMRA |  |
| Cox2 | Cox2 | F_CAACCCTGAATACCCAAGGA | [41] |
|  |  | R_GAAGCTTCTGATAGGCGGGA |  |
| Cox5 | Cox5 | F_CAGGAGCAAGCTTGAATGCG | [41] |
|  |  | R_TGGTATGACAACCCGTCATG |  |
| Cox18 | Cox18 | F_CGCAGACGAATTAGCCAATC | [41] |
|  |  | R_TTCGATGATCCGATGGCCTT |  |
| **Universal vertebrate**  16S ribosomal RNA | 16S | 16SA-CGCCTGTTTACCAAAAACAT | [42] |
|  |  | 16SB-CCGGTCTGAACTCAGATCACGT |  |
