# Supplementary material for: Detection of bacterial pathogens including potential new species in human head lice from Mali
Source: PLoS One. 2017 Sep 20;12(9):e0184621. doi: 10.1371/journal.pone.0184621 (PMC5606924; doi:10.1371/journal.pone.0184621)
Supplement: S2 Table — (RTF) [file pone.0184621.s002.rtf]

Source of infection	Status	Country	References	
Body lice 	Confirmed (natural vector) 	Worldwide 	[1,5,6]	
Head lice 	Suspected 	France, USA, Ethiopia, Congo RDC, Senegal, Nepal, Madagascar	[6,14,25,27,28,52]	
Kittens or cats 	Suspected 	France 	[48,49]	
Cat fleas	Suspected 	France 	[68]	
S2 Table. Source of B. quintana infection in humans. 
